# Preserved Efficacy of Intra-articular Autologous Protein Solution in Patients Aged Over 80 Years With Knee Osteoarthritis: A Comparison With Younger Counterparts

**DOI:** 10.7759/cureus.102175

**Published:** 2026-01-23

**Authors:** Yosuke Kaneko, Kazue Hayakawa, Sho Nojiri, Yasuo Niki, Nobuyuki Fujita

**Affiliations:** 1 Department of Orthopedic Surgery, Fujita Health University, Aichi, JPN; 2 Department of Orthopedic Surgery, Center for Musculoskeletal Surgery and Research, Fujita Medical Innovation Center Tokyo, Tokyo, JPN

**Keywords:** autologous protein solution, knee osteoarthritis/koa, koos score, kss score, older adult, regenerative therapy

## Abstract

Introduction: With ongoing societal aging, the demand for regenerative medicine has increased. Recently, reports demonstrating the efficacy of knee regenerative therapies have increased, although substantial proportions of these studies involved relatively younger populations. However, in Japan, where our hospital is located, it is common for patients well beyond the WHO-defined elderly age of 65 years to present with a desire for regenerative therapy. This retrospective cohort study investigated the utility of autologous protein solution (APS) knee joint injections by comparing their effects in patients aged 80 years and older and those younger than 80 years.

Methods: From July 2021 to August 2023, we administered single APS injections into the knee joints of patients diagnosed with osteoarthritis. Clinical assessment was conducted at baseline and at one and six months post-injection. Forty-one patients (44 knees) who completed all scheduled assessments were included in this analysis. Subjects were stratified into group E (≥80 years old) or group Y (<80 years old). Clinical evaluations included assessments of joint range of motion, inflammatory biomarkers (C-reactive protein), radiographic Kellgren-Lawrence classification, MRI findings including bone marrow edema, and standardized patient-reported outcome measures (Knee Injury and Osteoarthritis Outcome Score {KOOS} and Knee Society Score {KSS}), with KSS subdomain scores normalized to a 100-point scale.

Results: No distinct intergroup differences were observed for knee range of motion, blood test parameters, or MRI findings. Group E had the highest scores on the KOOS activities of daily living and quality of life subscales at one month post-APS administration; both subscale scores decreased at six months post-administration, whereas these subscale scores consistently increased over time in group Y. Compared with the findings in prior research reporting the minimum clinically important difference after platelet-rich plasma administration, the findings at six months after APS administration were superior for all KOOS subscales, excluding sports, in both groups. Concerning KSS scores, most subscale scores consistently increased throughout the follow-up period in both groups, whereas the expectation subscale score tended to decrease over time, likely reflecting the evolution of expectations after surgery.

Conclusions: Intra-articular APS injections demonstrated clinical efficacy in patients aged 80 years and older. When the analysis was limited to those who continued follow‑up at both one and six months after treatment, a tendency toward a shorter duration of effect was still observed, indicating the need for careful patient selection.

## Introduction

In recent years, the incidence of osteoarthritis has been increasing in tandem with global societal aging [1]. Currently, the estimated number of cases globally has reached approximately 374.7 million [2]. The progression of this condition leads to a decline in mobility attributable to knee joint pain [3], which in turn reduces activities of daily living (ADLs) [4], thereby increasing caregiving costs and exacerbating the societal burden [5]. An even more pronounced shift toward a super-aged society is anticipated in the coming years [6]. In Japan, where our medical institution is located, the average life expectancy has reached over 80 years for both men and women, far exceeding the age of 65 years defined by the WHO as individuals aged 65 years or older [7]. Reducing joint pain in older adults helps improve mobility, which in turn supports the extension of healthy life expectancy [8]. In the early stages of osteoarthritis, symptom control can be achieved through hyaluronic acid injections [9], orthotic therapy [10], and rehabilitation [11]. Although total knee arthroplasty is considered for advanced knee osteoarthritis [12], many patients are concerned about complications or simply elect to avoid invasive procedures [13]. Consequently, there is a growing trend toward the use of less invasive regenerative medical injections [14], such as intra-articular injections of platelet-rich plasma (PRP) or mesenchymal stem cells (MSCs) [15,16]. In particular, PRP has garnered attention, with increasing numbers of published clinical studies demonstrating its efficacy [17]. Subsequently, autologous protein solution (APS), characterized by a higher enrichment of anti‑inflammatory cytokines and leukocyte-derived proteins, compared with conventional PRP, has also been described [18,19]. Globally, multiple reports have demonstrated the effectiveness of APS administration, particularly in younger individuals with a mean age of 50-60 years [20]. Nonetheless, a significant number of older patients have advanced osteoarthritis, and these patients often have multiple comorbidities [21]. Therefore, a more detailed understanding of the effects of APS injections in older adults is desired. This study evaluated the outcomes of APS therapy for knee osteoarthritis by comparing outcomes between patients aged 80 years and older and those younger than 80 years, to enhance clinical understanding and guide future management strategies for APS injections.

## Materials and methods

Patients included in the evaluation

From July 2021 to August 2023, APS, prepared strictly according to the protocol specified in the APS kit (Warsaw, IN: Zimmer Biomet), was intra-articularly injected in patients with knee osteoarthritis. All injections were administered as a single dose. All patients who completed the clinical assessment questionnaires underwent clinical evaluations prior to the injection and at one and six months after the injection. Thereafter, these patients were assigned by age to group E (patients aged 80 years and older) or group Y (patients younger than 80 years).

Clinical assessment

Prior to and one and six months after APS administration, the Knee Injury and Osteoarthritis Outcome Score (KOOS) (covering the symptoms, pain, activities of daily living {ADL}, sports, and quality of life {QOL} subscales) and Knee Society Score (KSS) (covering the symptoms, satisfaction, expectation, and functional activities subscales) were assessed using patient questionnaires [22,23]. Each KSS subscale score was standardized to a 100-point scale. The following parameters were assessed prior to treatment and six months post-intervention: knee range of motion; C-reactive protein (CRP) levels to exclude inflammatory conditions and monitor any acute changes following APS administration; Kellgren-Lawrence (KL) classification based on plain radiographs; and MRI findings, with particular attention to the presence of bone marrow edema and other structural changes. The clinical evaluation was conducted by multiple examiners who attended to each patient. In the bilateral-knee cases, clinical evaluations were performed for each knee in the same manner as in the single‑knee cases. On the day of APS administration, joint aspiration was performed as needed in patients exhibiting intra-articular effusion of the knee. For one week following the procedure, the patients were instructed to avoid analgesics, including nonsteroidal anti-inflammatory drugs (NSAIDs), for temporary post-procedural pain, in accordance with the manufacturer’s recommendation. Alternatively, patients were advised to apply ice after confirming that no complications, such as infections, were present.

Statistical analysis

Statistical analyses were conducted using Welch’s t-test for between-group comparisons in each cohort. A p-value lower than 0.05 indicated statistical significance. All tests were two-sided, and no adjustments were made for multiple comparisons. Data are presented as the mean±standard deviation unless otherwise specified. Analyses were performed using Excel 2021 version 2506 (Redmond, WA: Microsoft Corp.).

## Results

Clinical observations and laboratory data

The study population included 41 individuals (44 knees) with a mean age of 75.9±9.7 years, including 28 women (30 knees) and 13 men (14 knees). Group E consisted of 18 patients (19 knees; including 14 knees in women {73.7%} and five knees in men {26.3%}) with a mean age of 84.3±4.7 years. The Kellgren-Lawrence (KL) classification in group E was as follows: KL2, one knee (5.3%); KL3, six knees (31.6%); and KL4, 12 knees (63.1%). Conversely, group Y consisted of 23 patients (25 knees, including 16 knees in women {64.0%} and nine knees in men {36.0%}) with an average age of 69.5±7.2 years. The KL classification in group Y was as follows: KL2, four knees (16.0%); KL3, 13 knees (52.0%); and KL4, eight knees (32.0%). These findings indicated a higher rate of advanced osteoarthritis in group E (Table 1).

**Table 1:** Demographic and clinical characteristics of patients aged 80 years and older versus those younger than 80 years. KL: Kellgren-Lawrence

Variables	Group E (≥80 years)	Group Y (<80 years)
Age (mean±SD)	84.3±4.7	69.5±7.2
Number of patients (n)	18	23
Number of knees	19	25
Knees (female)	14	16
Knees (male)	5	9
KL grade 2	1	4
KL grade 3	6	13
KL grade 4	12	8

Successful intra-articular injection was confirmed in all cases. No apparent adverse events were observed either locally at the affected site or systemically following APS administration. The mean knee range of motion was as follows: group E, 124.5±15.4° at baseline and 125.5±14.8° at six months post-APS administration; and group Y, 129.8±13.6° at baseline and 130.0±13.0° at six months post-APS administration. No statistically significant changes were observed within either group. Additionally, no consistent trends in CRP levels were observed before or after APS administration in either group. Although bone marrow lesions were sporadically observed on MRI, no consistent or specific changes, such as a reduction in signal intensity, were detected in either group before or after APS treatment.

KOOS score

**Figure 1:**
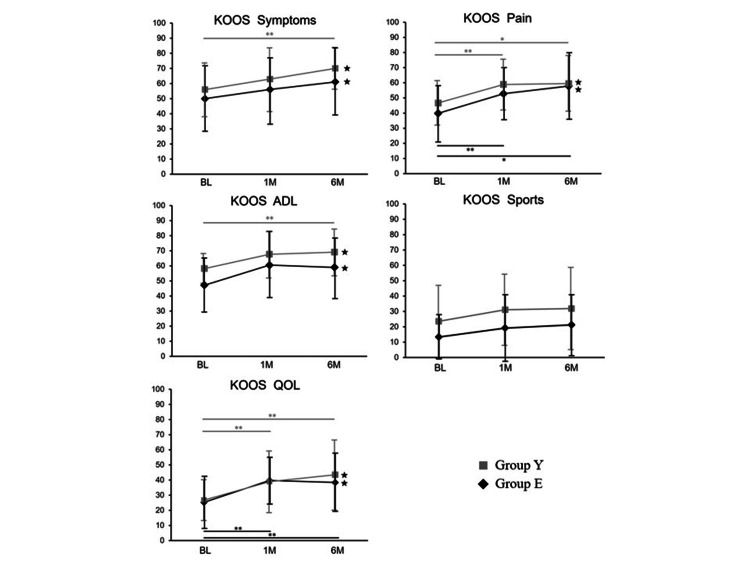
Meaningful improvements in KOOS scores were observed after APS treatment in both groups. *P<0.05 was statistically significant. **P<0.01 was statistically significant. ★Instances in the previously reported PRP data (MCID) were exceeded at six months post-treatment. The figure presents the trajectory of KOOS subscale scores from the pre-treatment baseline (BL) to the one-month (1M) and six-month (6M) follow-ups after intra-articular APS injection. Black diamonds indicate group E (≥80 years old), and gray squares indicate group Y (<80 years old). KOOS: Knee Injury and Osteoarthritis Outcome Score; MCID: minimum clinically important difference; PRP: platelet-rich plasma; APS: autologous protein solution

Before and one and six months after APS administration, the mean KOOS symptoms subscale scores were 50.0, 56.1, and 61.1, respectively, in group E, versus 56.0, 63.0, and 70.1, respectively, in group Y. Similarly, the KOOS pain subscale scores at these time points were 39.8, 52.8, and 57.9, respectively, in group E and 46.7, 58.9, and 59.5, respectively, in group Y. Regarding the KOOS ADL subscale, the scores before and one, and six months after APS administration were 47.2, 60.5, and 59.0, respectively, in group E and 58.2, 67.6, and 69.1, respectively, in group Y, whereas the KOOS sports subscale scores at these time points were 13.4, 19.2, and 21.3, respectively, in group E and 23.6, 31.2, and 31.9, respectively, in group Y. The KOOS QOL subscale scores before and one and six months after APS administration were 25.3, 40.5, and 38.5, respectively, in group E and 26.7, 39, and 43.5, respectively, in group Y. Thus, the KOOS ADL and QOL subscale scores in group E were highest at one month post-APS administration and subsequently decreased at six months post-therapy, whereas these values consistently increased over time in group Y (Figure 1). When compared to reports presenting the minimum clinically important difference (MCID) for platelet-rich plasma (PRP) intra-articular injections, the results at six months post-treatment exceeded this threshold for all KOOS subscales, excluding sports, in both groups [24].

KSS score

**Figure 2:**
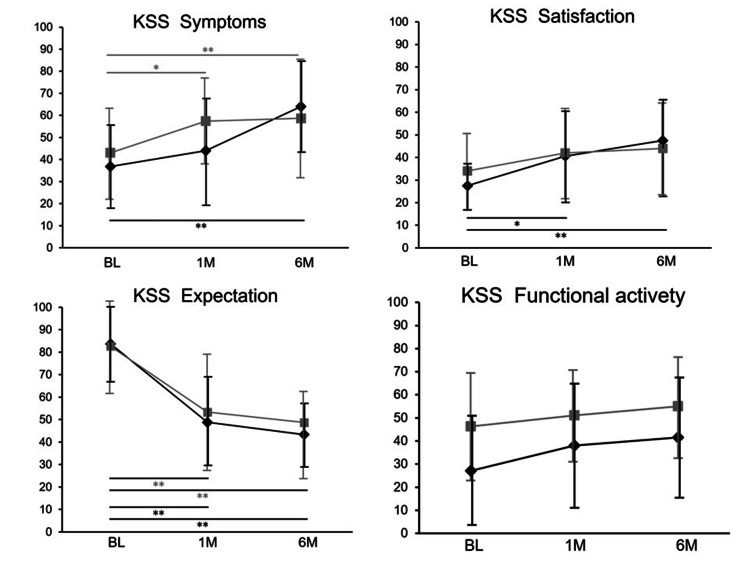
KSS subscales, excluding the expectation subscale, progressively improved over time in both groups. *P<0.05 was statistically significant. **P<0.01 was statistically significant. This figure illustrates the changes in each KSS subscale from baseline (BL) to one month (1M) and six months (6M) after intra-articular APS injection. All subscales are presented on a 100-point scale. Black diamonds indicate group E (≥80 years old), and gray squares indicate group Y (<80 years old). KSS: Knee Society Score; APS: autologous protein solution

Before and one and six months following APS administration, the mean KSS symptoms subscale scores were 9.2, 11.0, and 16.0, respectively, in group E, and 10.8, 14.4, and 14.7, respectively, in group Y. The mean KSS satisfaction subscale scores at these time points were 11.0, 16.7, and 20.2, respectively, in group E, and 13.6, 16.4, and 17.0, respectively, in group Y, whereas the KSS Expectation subscale scores were 13.1, 7.3, and 6.5, respectively, in group E, and 11.9, 7.8, and 7.3, respectively, in group Y. The KSS functional activity subscale scores before and one and six months after APS administration were 27.1, 36.3, and 36.8, respectively, in group E, and 46.3, 48.9, and 54.5, respectively, in group Y. Excluding the KSS Expectation subscale score, which appeared to decline over time because of the nature of the assessment itself, all other subscale scores consistently increased over time in both groups (Figure 2).

## Discussion

Previous reports on APS treatment

Clinical studies have reported the intra-articular administration of regenerative medicine for osteoarthritis of the knee, such as PRP injection and mesenchymal stem cells (MSCs) derived from adipose tissue, synovium, and other origins, and their benefits are increasingly being recognized [25]. APS is derived from PRP containing a high density of platelets, and it possesses a diverse and abundant profile of growth factors and anti-inflammatory mediators [26]. Several reports demonstrated the safety and efficacy of APS injections. van Drump et al. reported significant clinical effects in patients with mild-to-moderate knee osteoarthritis [20]. Their study included a population with a mean age of 57.5 years, and significant improvements were observed across all Western Ontario and McMaster Universities Osteoarthritis Index (WOMAC) subscales. The responder rate was assessed by applying the Outcome Measures in Rheumatology-Osteoarthritis Research Society International (OMERACT-OARSI) criteria, demonstrating greater than 60% improvement at four weeks post-treatment and greater than 70% improvement at 12 and 26 weeks post-treatment [27]. King et al. were the first to report strong correlations of the white blood cell (WBC) count and interleukin (IL) concentration with the WOMAC score. A high responder rate was observed when IL-1RA/IL-1β ratio was greater than 1,000, and the WBC count was greater than 30,000 [28]. van Drumpt et al. described the greater clinical effectiveness of APS in patients with mild cartilage damage than in those with severe damage. The subjects in these studies had a mean age in the late 50s, representing relatively younger patients [19]. Kuwasawa et al. also analyzed the responder rate with a focus on the KOOS and OMERACT-OARSI criteria, reporting higher response rates (58%) among patients with early-stage knee osteoarthritis (KL grade 2), with the response rate declining with disease progression [29].

Based on previously published reports from various countries, APS therapy has greater efficacy in relatively younger individuals aged 50-60 years and in patients with early-stage knee osteoarthritis corresponding to KL classification grade 2. At our institution, patients in their 50s and 60s tend to receive APS therapy primarily for pain relief in the early stages of knee osteoarthritis, whereas older patients are more likely to undergo APS treatment even in more advanced stages, including moderate-to-severe osteoarthritis. Consequently, we decided to evaluate patients’ clinical courses by dividing the cohort based on an age threshold of 80 years.

Because this was an observational study that mechanically divided patients at the 80-year cutoff rather than using propensity-matched cohorts, it is natural, and indeed unavoidable, that various characteristics beyond age differed between the two groups. Nevertheless, these disparities are considered to meaningfully reflect the distinct profiles and contextual backgrounds of groups E and Y.

Clinical course characteristics and considerations following APS injection in groups aged 80 years and older versus those younger than 80 years

Following APS injection, improvements in the KOOS ADL and QOL subscales were observed at one month post-treatment in group E; however, these scores were lower after six months. Conversely, group Y exhibited consistent improvements across all KOOS subscales over time, with progressive enhancement observed at one and six months post-intervention. This discrepancy might be partially attributable to differences in mean age between the groups, as well as the higher proportion of patients with progressive knee osteoarthritis in group E. Group E patients were older, and their baseline clinical scores were lower than those of group Y patients. Intriguingly, the six-month KSS satisfaction score was higher in group E compared with group Y. Based on the available data, this paradox might be attributable to the fact that group E patients initially exhibited more severe symptoms and had relatively low expectations for improvement. Consequently, the observed clinical improvement might have exceeded their anticipated outcomes, leading to a heightened perception of therapeutic benefit. The trends elucidated in the present study provide novel insights that should be remembered when considering APS therapy for older patients with knee osteoarthritis. Although clinical improvement can generally be expected, the comparatively shorter duration of effect in this age group relative to that in patients younger than 80 years could be an important consideration in selecting appropriate treatment options.

Limitations

In this study, clinical evaluations were limited to patients who could be assessed at the follow-up periods of one and six months after the single APS intervention, which constitutes a limitation of the research. We compared groups aged 80 years and above with those under 80 years. Ideally, the comparison should be performed using propensity matching or other appropriate control methods; however, since the backgrounds of each group differ, this is practically not feasible. Therefore, the analysis is limited to considerations based on the populations in which follow‑up was possible after a single administration of APS to each group with differing backgrounds.

## Conclusions

APS knee joint injections were administered to older adult patients, and clinical outcomes were evaluated at one and six months post-intervention. Although an overall trend of improvement was observed in both groups, an earlier decline in clinical scores, as evaluated by KOOS and KSS, was observed in older patients. Although further research is warranted, these findings might represent a useful reference when considering APS therapy, as well as other conservative or surgical treatments, for older patients with knee osteoarthritis.

## References

[1] Robert H, Brophy RH, Fillingham YA (2022). AAOS clinical practice guideline summary: management of osteoarthritis of the knee (nonarthroplasty), third edition. J Am Acad Orthop Surg.

[2] Ren JL, Yang J, Hu W (2025). The global burden of osteoarthritis knee: a secondary data analysis of a population-based study. Clin Rheumatol.

[3] Dainese P, Wyngaert KV, De Mits S, Wittoek R, Van Ginckel A, Calders P (2022). Association between knee inflammation and knee pain in patients with knee osteoarthritis: a systematic review. Osteoarthr Cartil.

[4] Gupta A, Maffulli N (2009). Growth factor concentrate (GFC) for the management of osteoarthritis of the knee: a systematic review. Indian J Orthop.

[5] Mallio CA, Bernetti C, Agostini F (2022). Advanced MR imaging for knee osteoarthritis: a review on local and brain effects. Diagnostics.

[6] Punnoose A, Claydon-Mueller LS, Weiss O, Zhang J, Rushton A, Khanduja V (2023). Prehabilitation for patients undergoing orthopedic surgery: a systematic review and meta-analysis. JAMA Netw Open.

[7] Tokudome S, Hashimoto S, Igata A (2016). Life expectancy and healthy life expectancy of Japan: the fastest graying society in the world. BMC Res Notes.

[8] Nakamura T, Koga H (2024). Review of the development of meniscus centralization. Curr Rev Musculoskelet Med.

[9] Cooper C, Rannou F, Richette P (2017). Use of intraarticular hyaluronic acid in the management of knee osteoarthritis in clinical practice. Arthritis Care Res.

[10] Sprouse RA, McLaughlin AM, Harris GD (2018). Braces and splints for common musculoskeletal conditions. Am Fam Physician.

[11] Tore NG, Oskay D, Haznedaroglu S (2022). The quality of physiotherapy and rehabilitation program and the effect of telerehabilitation on patients with knee osteoarthritis. Clin Rheumatol.

[12] Gelber AC (2024). Knee osteoarthritis. Ann Intern Med.

[13] Ivirico J, Bhattacharjee M, Kuyinu E, Nair LS, Laurencin CT (2017). Regenerative engineering for knee osteoarthritis treatment: biomaterials and cell-based technologies. Engineering.

[14] Zhang JY, Xiang XN, Yu X (2024). Mechanisms and applications of the regenerative capacity of platelets-based therapy in knee osteoarthritis. Biomed Pharmacother.

[15] Filardo G, Previtali D, Napoli F, Candrian C, Zaffagnini S, Grassi A (2021). PRP injections for the treatment of knee osteoarthritis: a meta-analysis of randomized controlled trials. Cartilage.

[16] Xiang XN, Zhu SY, He HC, Yu X, Xu Y, He CQ (2022). Mesenchymal stromal cell-based therapy for cartilage regeneration in knee osteoarthritis. Stem Cell Res Ther.

[17] Szwedowski D, Szczepanek J, Paczesny Ł, Zabrzyński J, Gagat M, Mobasheri A, Jeka S (2021). The effect of platelet-rich plasma on the intra-articular microenvironment in knee osteoarthritis. Int J Mol Sci.

[18] Woodell-May J, Steckbeck K, King W (2021). Potential mechanism of action of current point-of-care autologous therapy treatments for osteoarthritis of the knee - a narrative review. Int J Mol Sci.

[19] Angadi DS, Macdonald H, Atwal N (2020). Autologous cell-free serum preparations in the management of knee osteoarthritis: what is the current clinical evidence?. Knee Surg Relat Res.

[20] van Drumpt RA, van der Weegen W, King W, Toler K, Macenski MM (2016). Safety and treatment effectiveness of a single autologous protein solution injection in patients with knee osteoarthritis. Biores Open Access.

[21] Palazzo C, Nguyen C, Lefevre-Colau MM, Rannou F, Poiraudeau S (2016). Risk factors and burden of osteoarthritis. Ann Phys Rehabil Med.

[22] Roos EM, Lohmander LS (2003). The Knee injury and osteoarthritis outcome score (KOOS): from joint injury to osteoarthritis. Health Qual Life Outcomes.

[23] Mirahmadi A, Hosseini-Monfared P, Amiri S, Taheri F, Farokhi M, Noshahr RM, Kazemi SM (2023). Cross‑cultural adaptation and validation of the Persian version of the new Knee Society Knee Scoring System (KSS). J Orthop Surg Res.

[24] Boffa A, Andriolo L, Franceschini M (2021). Minimal clinically important difference and patient acceptable symptom state in patients with knee osteoarthritis treated with PRP injection. Orthop J Sports Med.

[25] Dulic O, Rasovic P, Lalic I (2021). Bone marrow aspirate concentrate versus platelet rich plasma or hyaluronic acid for the treatment of knee osteoarthritis. Medicina.

[26] Wasai S, Sato M, Maehara M (2020). Characteristics of autologous protein solution and leucocyte-poor platelet-rich plasma for the treatment of osteoarthritis of the knee. Sci Rep.

[27] Pham T, van der Heijde D, Altman RD (2004). OMERACT-OARSI initiative: Osteoarthritis Research Society International set of responder criteria for osteoarthritis clinical trials revisited. Osteoarthr Cartil.

[28] King W, van der Weegen W, Van Drumpt R, Soons H, Toler K, Woodell-May J (2016). White blood cell concentration correlates with increased concentrations of IL-1RA and improvement in WOMAC pain scores in an open-label safety study of autologous protein solution. J Exp Orthop.

[29] Kuwasawa A, Okazaki K, Noda K, Nihei K (2023). Clinical results of autologous protein solution injection for knee osteoarthritis with severe disease grade is inferior to mild or moderate grade. Sci Rep.

